# Predictive value of neutrophil gelatinase-associated lipocalin in children with acute kidney injury: A systematic review and meta-analysis

**DOI:** 10.3389/fped.2023.1147033

**Published:** 2023-03-27

**Authors:** Zhuan Zou, Bin Chen, Fajuan Tang, Xihong Li, Dongqiong Xiao

**Affiliations:** ^1^ Department of Emergency, West China Second University Hospital, Sichuan University, Chengdu, China; ^2^ Key Laboratory of Birth Defects and Related Diseases of Women and Children, Ministry of Education, Sichuan University, Chengdu, China

**Keywords:** predictive value, neutrophil gelatinase-associated lipocalin, children, acute kidney injury, meta-analysis

## Abstract

**Purpose:**

Neutrophil gelatin lipase carrier protein (NGAL) has been used as an early biomarker to predict acute kidney injury (AKI). However, the predictive value of NGAL in urine and blood in children with acute kidney injury in different backgrounds remains unclear. Therefore, we conducted this systematic review and meta-analysis to explore the clinical value of NGAL in predicting AKI in children.

**Methods:**

Computerized databases were searched for relevant the studies published through August 4th, 2022, which included PUBMED, EMBASE, COCHRANE and Web of science. The risk of bias of the original included studies was assessed by using the Quality Assessment of Studies for Diagnostic Accuracy (QUADA-2). At the same time, subgroup analysis of these data was carried out.

**Results:**

Fifty-three studies were included in this meta-analysis, involving 5,049 patients, 1,861 of whom were AKI patients. The sensitivity and specificity of blood NGAL for predicting AKI were 0.79 (95% CI: 0.69–0.86) and 0.85 (95% CI: 0.75–0.91), respectively, and SROC was 0.89 (95% CI: 0.86–0.91). The sensitivity and specificity of urine NGAL for predicting AKI were 0.83 (95% CI: 0.78–0.87) and 0.81 (95% CI: 0.77–0.85), respectively, and SROC was 0.89 (95% CI: 0.86–0.91). Meanwhile, the sensitivity and specificity of overall NGAL (urine and blood NGAL) for predicting AKI in children were 0.82 (95% CI: 0.77–0.86) and 0.82 (95% CI: 0.78–0.86), respectively, and SROC was 0.89 (95% CI: 0.86–0.91).

**Conclusion:**

NGAL is a valuable predictor for AKI in children under different backgrounds. There is no significant difference in the prediction accuracy between urine NGAL and blood NGAL, and there is also no significant difference in different measurement methods of NGAL. Hence, NGAL is a non-invasive option in clinical practice. Based on the current evidence, the accuracy of NGAL measurement is the best at 2 h after cardiopulmonary bypass (CPB) and 24 h after birth in asphyxiated newborns.

**Systematic Review Registration:**

https://www.crd.york.ac.uk/prospero/, identifier: CRD42022360157.

## 1. Background

At present, acute kidney injury is a great challenge for both adults and children in clinical practice, which occur more frequently in patients with cardiac surgery or in critically-ill children (1, 2). AKI affects almost 20%–50% of children admitted to the pediatric intensive care units (3, 4). However, there has been no recognized unified tool for the early prediction of AKI up to now, and many researchers have introduced machine learning into the field (5–7). Machine learning and single-factor prediction can complement each other. Efficient predictors are the key to improving the accuracy of machine learning predictions. However, for a single predictor, the accuracy of AKI diagnosis can be combined with other factors by using machine learning. Thus, improvement requires the combination of various effective predictors. Several studies have explored various predictors of AKI, including kidney injury molecule-1 (KIM-1) (8), liver lipase-binding protein (L-FABP) (8, 9), NGAL (10, 11), tissue inhibitor of metalloproteinase-2 (TIMP-2) and insulin-like growth factor binding protein 7 (IGFBP7) (12–14), etc.

Recent studies have found that NGAL seems to have good predictive ability for AKI, but its predictive ability for AKI in children in different backgrounds is still unclear, and there is no evidence-based evidence to explore whether it is derived from blood or urine. To evaluate the accuracy of NGAL in predicting AKI occurrence in different backgrounds, we conducted this systematic review and meta-analysis and aimed to provide a reference for the development or update of assessment tools for AKI in children in clinical practice.

## 2. Methods

The systematic review was implemented strictly according to PRISMA-2020 (Preferred Reporting Items for Systematic Reviews and Meta-Analyses) and prospectively registered on PROSPER (ID: CRD42022360157).

### 2.1. Literature search strategies

We systematically searched Pubmed, Cochrane, Embase, and Web of Science databases for relevant studies published between 2005 and 2022. The age of participants ranged from 0 to 18. The search strategy adopted the combination of subject headings and free words. No restrictions were imposed on regions or languages. The detailed searching strategies are shown in Supplementary Table S1.

### 2.2. Inclusion and exclusion criteria

#### 2.2.1. Inclusion criteria

1. Studies including pediatric patients with complete records about NGAL;
2. Studies including high levels of NGAL without limitation to the obvious cutoff value;
3. Studies whose original types were RCTs, cohort studies, or case-control studies.

#### 2.2.2. Exclusion criteria

1. When it was not possible to distinguish whether NGAL was present in children or adults in a study, we considered excluding the study;
2. If a study had a large number of missing values about NGAL, and the missing values were not effectively processed, the study was excluded;
3. One of the following outcome indicators cannot be extracted directly or indirectly: sensitivity, specificity, diagnostic four-table, OR value.

### 2.3. Study selection and data extraction

The retrieved studies were imported into EndNote, and the titles and abstracts were screened to include the initial studies that met the inclusion criteria. After downloading and reviewing the full text of all potentially eligible studies, we included the eligible studies against the same eligibility criteria in our systematic review. We developed a standard data extraction spreadsheet before data extraction. Extraction content included the title, the first author's name, publication year, study type, NGAL source and measurement methods, AKI occurrence backgrounds and time, NGAL optimal cut-off threshold, the number of patients with AKI and the total number of study cases, sensitivity and specificity. The above literature screening and data extraction were independently carried out by two investigators (ZZ and CB) and cross-checked after completion. Any disagreements would be resolved by a third investigator (X-DQ).

### 2.4. Quality assessment

The quality and risks of bias of the initially included studies were assessed by two researchers independently (ZZ and CB) with the help of the Quality Assessment of Diagnostic Accuracy Studies (QUADAS-2) tool (15). This tool consists of four key domains: patient selection, index test, reference standard, flow and timing. The risks of bias in all studies were assessed according to these four domains and the applicability concerns were evaluated according to the former three: these areas were marked as the following responses: “Yes”, “No” or “Unclear”. For the evaluation of the risk of bias, if all of the questions in a domain were marked with “Yes” responses, the domain would be ranked as a low risk of bias. Any questions in a domain marked with “No” responses suggested a high risk of bias.

### 2.5. Statistical analysis

We conducted a meta-analysis in which a bivariate mixed effects model in the “Midas” command of Stata 15.0 (StataCorp LLC, College Station, TX) was used to assess sensitivity (SEN), specificity (SEP), positive likelihood ratio (PLR), negative likelihood ratio (NLR), and diagnostic odds ratio (DOR). We calculated the point estimates with 95% confidence intervals (95% CI). A summary receiver operating characteristic curve (SROC) was drawn and the area under the curve (AUC) at 95% CI was calculated. Deek's funnel plot was used to evaluate the publication bias, and the Q statistic and I^2^ statistic were used to test for heterogeneity. *P* < 0.05 was considered statistically significant.

## 3. Results

### 3.1. Literature screening results

**Figure 1:**
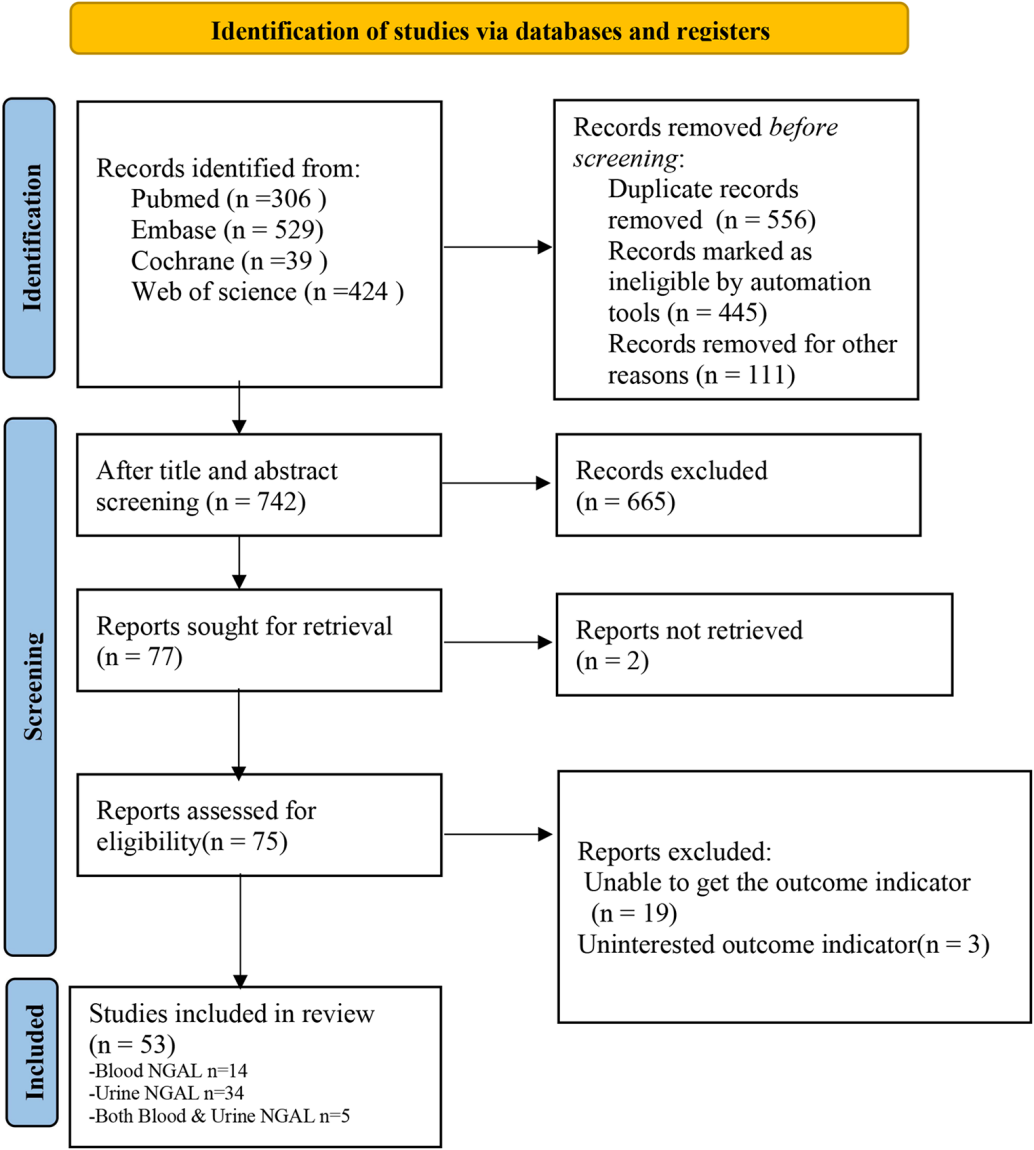
Flow chart of literature screening. AKI acute kidney injury, area under the AUC curve, NGAL neutrophil gelatin release-associated lipoprotein.

Overall, 1,298 relevant studies were retrieved from the database, of which 556 duplicates were excluded. After screening the titles and abstracts of the remaining 742 studies, 665 studies were excluded. After screening the full text, 24 studies were excluded and 53 met the eligibility criteria (10, 11, 16–65). The flowchart of the literature screening method is shown in Figure 1.

### 3.2. Study characteristics

Of the 53 included studies published between 2005 and 2022, which included 5,048 patients, 1,861 patients had AKI, with an incidence rate of 36.8%. Among them, 34 studies (10, 11, 16–18, 20–22, 24, 27–29, 31–34, 39, 42–45, 47, 48, 52, 53, 55–58, 61–65) measured urine NGAL, 14 studies (24, 26, 30, 36–38, 41, 46, 49–51, 54, 59, 60) measured blood NGAL, and 5 studies (19, 25, 35, 40, 42) measured blood and urine simultaneously. The included studies were conducted in 20 countries worldwide, including 11 studies in European countries (19, 20, 30, 33–36, 38, 54, 58, 65), 12 studies in the United States (10, 11, 32, 40, 42, 45, 49, 50, 53, 61, 62, 66), 20 studies in Asian countries (16, 18, 22, 24, 25, 27–29, 31, 33, 39, 41, 43, 44, 48, 51, 52, 55, 56, 59), 9 studies in African countries (24, 37, 46, 47, 49, 57, 60, 63, 64), and 1 study from Canada (17). Two studies were multi-centered (26, 33), and the remaining studies were single-centered. The included studies covered various backgrounds of AKI. Among them, 15 studies reported AKI occurred after CPB surgery (11, 19, 21, 24, 33, 36, 40, 42, 46, 50, 53, 56, 58, 65, 66), 12 studies reported AKI occurred in asphyxiated neonates (22, 28, 30, 31, 35, 37–39, 47, 51, 54, 57), and the remaining 26 studies reported AKI occurred in children hospitalized for a variety of diseases, including sepsis, burns, solid tumors, post-liver transplantation, nephrotoxic drug use, imaging, and preterm, neonatal congenital heart disease, neonatal Hyperbilirubinemia, neonatal general surgery, etc. The diagnostic criteria for AKI used in the original studies varied and were mainly divided into the following three: pediatric risk, injury, failure, loss, final and end stage (PRIFLE) (67), Acute Kidney Injury Network (AKIN) (68) and Kidney Disease: Improving Global Outcomes (KDIGO) (69). The most frequently used measurement method for blood or urine NGAL in the included studies was enzyme-linked immunosorbent assay (ELISA), and 7 studies used immunoturbidimetry (17, 19, 22, 51, 54, 58, 61), 2 studies used chemiluminescence microparticle immunoassay (CMIA) (20, 33), and 1 study used Fluorescence-based immunoassay (IFA) (36). The main characteristics and details of all included study are shown in Table 1, and the quality assessment of the 53 included studies is shown in Table 1.

**Table 1:** Characteristics of included studies of AKI in children.

No	Author	Study Design	Country	NGAL source	Determination method	Diagnostic criteria	Pathogenesis background (y)	Events	Sample size	Hospital departments	Boy's Proportion	Age stage	Sampling Time (hours)
1	Jaya Mishra	Cohort study	USA	Urine/Blood	ELISA	pRIFLE	CPB	20	71	Admission	0.63	Children	2 (after CPB)
2	Catherine L Dent	Cohort study	USA	Blood	ELISA	pRIFLE	CPB	45	120	Admission	0.53	Children	2 (after CPB)
3	Michael Zappitelli	Cohort study	USA	Urine	ELISA	pRIFLE	Critically-ill children	106	140	PICU	0.54	Children	48 (after admission)
4	Michael Bennett	Cohort study	USA	Urine	ELISA	pRIFLE	CPB	99	196	Admission	0.53	Children	2 (after CPB)
5	Derek S. Wheeler	Cohort study	USA	blood	ELISA	pRIFLE	Sepsis	22	143	PICU	0.65	Children	24(after admission)
6	Catherine D	Cohort study	USA	Urine	ELISA	pRIFLE	CPB	60	220	Admission	0.48	Children	2 (after CPB)
7	Catherine D	Cohort study	USA	Urine/blood	ELISA	pRIFLE	CPB	112	373	Admission	0.53	Children/Neonates	2 (after CPB)
8	O.G. El-Farghali	Case-control	Egypt	blood	ELISA	AKIN	Critically-ill Neonates	34	60	NICU	0.57	Neonates	48 (after admission)
9	Fatina I. Fadel	Cohort study	Egypt	blood	ELISA	pRIFLE	CPB	19	40	PICU	0.50	Children	2 and 12 (after CPB)
10	Zaccaria Ricci	Case-control	Italy	blood	IFA	pRIFLE	CPB	90	160	PICU	0.55	Children	24 (after admission)
11	Kosmas Sarafidis	Case-control	Greece	Urine/Blood	ELISA	KDIGO	Asphyxiated Neonates	8	13	NICU	0.77	Neonates	24 (after birth)
12	S. B. Hoffman	Cohort study	USA	Urine	ELISA	pRIFLE	Critically-ill Neonates	15	35	NICU	0.60	Neonates	24 (after admission)
13	Amira peco-Antic	Cohort study	Serbia	Urine	ELISA	pRIFLE	CPB	18	112	Admission	0.58	Children	24 (after CPB)
14	N. E. Raggal	Cohort study	Egypt	blood	ELISA	KDIGO	Asphyxiated Neonates	12	30	NICU	0.57	Neonates	6 (after birth)
15	Stefanie Seitz	Cohort study	Germany	Urine	CMIA	pRIFLE	CPB	76	139	Admission	0.55	Children	2 (after CPB)
16	Doaa Mohammed Youssef	Cohort study	Egypt	Blood	ELISA	pRIFLE	Critically-ill Neonates	13	60	PICU	0.47	Children	at the time of admission
17	Jianyong Zheng	Cohort study	China	Urine	ELISA	AKIN	CPB	29	58	Admission	0.67	Children	4 (after CPB)
18	A.J.Alcaraz	Cohort study	Spain	Urine	Immunoturbidimetry	pRIFLE	CPB	42	106	PICU	0.58	Children	1 (after CPB)
19	Kosmas Sarafidis	Case-control	Greece	Urine	ELISA	pRIFLE	Preterm neonates	11	22	NICU	0.50	Neonates	at the time of admission
20	Yilmaz Tabel	Case-control	Turkey	Urine	ELISA	pRIFLE	Preterm neonates	6	50	NICU	0.58	Neonates	24 (after admission)
21	Sevgi Yavuz	Cohort study	Turkey	Urine/Blood	ELISA	pRIFLE	burned children	6	22	PICU	0.55	Children	at the time of admission
22	M. A. Almalky	Case-control	Egypt	Urine	ELISA	pRIFLE	solid tumors	14	30	Admission	0.50	Children	at the end of the chemotherapy protocol
23	Farida Essajee	Cohort study	Kenya	Urine	ELISA	KDIGO	Asphyxiated Neonates	60	108	NICU	0.60	Neonates	24 (after birth)
24	B. Pejovic	Case-control	Serbia	Blood	ELISA	KDIGO	Asphyxiated Neonates	73	108	NICU	0.60	Neonates	2 and 4 (after birth)
25	Piotr Surmiak	Cohort study	Poland	Blood	ELISA	AKIN	Heart syndrome	8	21	NICU	0.38	Neonates	During childbirth
26	Alexandra JM Zwiers	Cohort study	TheNetherlands	Urine	CMIA	pRIFLE	Critically-ill Neonates	35	100	NICU	0.66	Neonates	6 (after admission)
27	Mohammed Farouk M	Cohort study	Egypt	Blood	ELISA	pRIFLE	Sepsis	30	65	PICU	0.49	Children	24 (after admission)
28	C. Channanayaka	Cohort study	India	Blood	Immunoturbidimetry	KDIGO	Asphyxiated Neonates	3	30	NICU	0.50	Neonates	6 (after birth)
29	Mehmet Yekta Oncel	Case-control	Turkey	Urine	ELISA	AKIN	Asphyxiated Neonates	15	41	NICU	0.56	Neonates	24 (after birth)
30	M. Hanna	Case-control	USA	Urine	Immunoturbidimetry	KDIGO	Critically-ill Neonates	25	45	NICU	0.44	Neonates	24 (after admission)
31	V. Tanigasalam	Cohort study	India	Urine	ELISA	AKIN	Asphyxiated Neonates	55	120	NICU	0.57	Neonates	6 (after birth)
32	M. Baumert	Cohort study	Poland	Blood	Immunoturbidimetry	AKIN	Asphyxiated Neonates	8	43	NICU	0.53	Neonates	24 (after birth)
33	A. T. Elmas	Case-control	Turkey	Urine	ELISA	KDIGO	Preterm neonates	13	64	NICU	0.48	Neonates	24 (after birth)
34	YU’E SONG	Case-control	China	Urine	ELISA	pRIFLE	Asphyxiated Neonates	48	93	NICU	0.56	Neonates	24 and 48 (after birth)
35	Neamatollah Ataei	Cohort study	Iran	Urine	ELISA	pRIFLE	Admission	13	96	Admission	0.56	Children	within 6 h of admission
36	Jameela Abdulaziz Kari	Cohort study	Kingdom of Saudi Arabia	Urine	ELISA	pRIFLE	Critically ill children	22	40	PICU	0.45	Children	at the time of admission
37	Kathleen G. Brennan	Cohort study	USA	Urine	ELISA	KDIGO	CPB	17	76	NICU	0.70	Neonates	4 (after CPB)
38	Dana Y. Fuhrman	Cohort study	USA	Urine	ELISA	KDIGO	undergoing liver transplantation	6	16	PICU	0.56	Children	within 6 h after transplant
39	Nastaran Khosravi	Case-control	Iran	Urine	ELISA	KDIGO	Critically-ill Neonates	75	156	NICU	0.48	Neonates	at the time of admission
40	Hui LI	Cohort study	China	Blood	ELISA	KDIGO	Nephrotoxic medication	20	62	Admission	0.53	Children	24 (after admission)
41	Zhen Wang	Cohort study	China	Urine	ELISA	KDIGO	Neonatal Hyperbilirubinemia-related	27	196	NICU	0.48	Neonates	24 (after admission)
42	Fumiya Yoneyama	Case-control	Japan	Urine	ELISA	KDIGO	CPB	47	103	NICU	0.52	Children	at the time of admission
43	Ying Zhang	case-control	China	Urine	Immunoturbidimetry	KDIGO	Asphyxiated Neonates	37	110	NICU	0.67	Neonates	24 (after birth)
44	Yamini Agarwal	Cohort study	India	Blood	ELISA	pRIFLE	CI-AKI	35	100	PICU	0.60	Children	at 6 h after contrast
45	Walaa H Ali	Case-control	Egypt	Urine	ELISA	KDIGO	Asphyxiated Neonates	18	45	NICU	0.62	Neonates	6 (after birth)
46	Bhattacharjee Aniruddha	Cohort study	India	Urine	ELISA	pRIFLE	CPB	42	76	PICU	0.67	Children	6 (after CPB)
47	Nugroho Setia Budi	Case-control	Indonesia	Urine	ELISA	KDIGO	Sepsis	17	30	PICU	0.70	Children	at the time of admission
48	Cara L. Slagle	Cohort study	USA	Urine	ELISA	KDIGO	general surgical procedures	33	141	NICU	0.55	Neonates	24 (after general surgical)
49	Vijai Williams	Case-control	India	Urine	ELISA	KDIGO	DKA	35	66	PICU	0.50	Children	48 (after admission)
50	Anthony Batte	Case-control	Uganda	Urine	ELISA	KDIGO	SCA	35	185	Admission	0.58	Children	2 (after admission)
51	Slobodan Galić	Cohort study	Croatia	Urine/Blood	Immunoturbidimetry	KDIGO	CPB	25	100	Admission	0.51	Children	6 and 12 (after CPB)
52	Sudeep K Kapalavai	Cohort study	India	Urine	ELISA	pRIFLE	Critically ill Children	59	130	PICU	0.55	Children	6 (after admission)
53	Kelly R. McMahon	Cohort study	Canada	Urine	ELISA	KDIGO	Nephrotoxic medication	46	156	Admission	0.50	Children	at early cisplatin visits
								22	127		0.51		at late cisplatin visits

1. children are younger persons aged > 28 days, Neonates are babies born 0–28 days. 2. PRIFLE, pediatric risk, injury, failure, loss, final and end stage; AKIN, Acute Kidney Injury Network; KDIGO, Kidney Disease: Improving Global Outcomes.

### 3.3. Quality assessment

**Figure 2:**
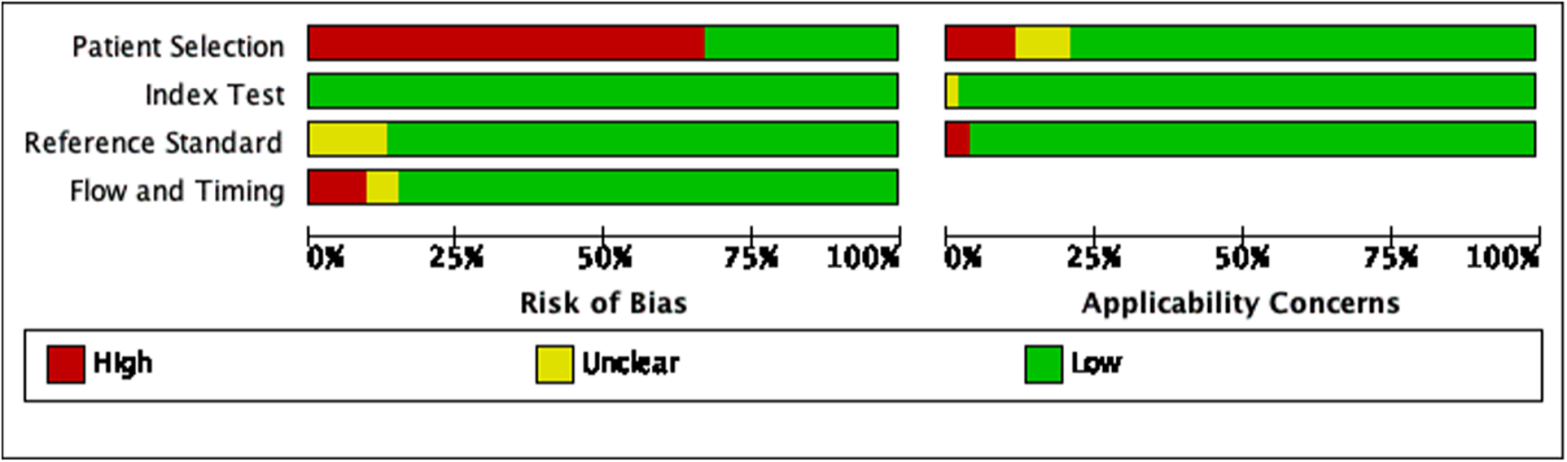
Risk of bias graph.

Of all included studies, 19 studies used case-control studies (11, 16, 19, 22, 24, 29, 31, 34–36, 38, 39, 43, 48, 49, 52, 57, 61, 64), and the remaining 34 used cohort studies; 7 studies did not report whether the interpretation of the gold standard results was blinded (26, 29, 38, 43, 45, 48, 57); 4 studies did not report whether there was an appropriate time interval between the trial under review and the gold standard (10, 32, 49, 64), and 3 studies had an inappropriate time interval between the trial under review and the gold standard (19, 31, 65); patients in 2 studies received two or more different gold standards (42, 56); in the matching of the included patients' backgrounds and evaluation questions, 3 studies had a high risk of bias because of the special NGAL measurement methods (20, 33, 36)and 1 study had a high risk of bias because the overall ages of the patients were too old (62). Two studies have a high risk of bias in the evaluation of the applicability of the gold standard (31, 56). The specific evaluation results are shown in Figure 2 and Supplementary Figure S1.

### 3.4. Meta-analysis

#### 3.4.1. Predictive accuracy of overall NGAL for AKI in children

Of these 53 studies, 9 studies were multiarm predictive diagnostic studies (17, 19, 25, 31, 35, 38, 40, 42, 46), thus equaling 64 for the total number of meta-analyses. A bivariate effect model was used to perform a meta-analysis of NGALto predict AKI in children, with a sensitivity of 0.82 (95% CI: 0.77–0.86), specificity of 0.82 (95% CI: 0.78–0.86), and an SROC of 0.89 (95% CI: 0.86–0.91), DOR 21 (95% CI: 14–31), PLR (Positive likelihood ratio) 4.6 (95% CI: 3.6–5.8), NLR (Negative likelihood ratio) 0.22 (95% CI: 0.17–0.29). The results of the subgroup analysis can be seen in Table 2.

**Table 2:** Meta-analysis (sensitivity analysis) of prediction of AKI by overall NGAL.

Subgroup	Levels	Number	Sensitivity (95% CI)	Specificity (95% CI)	SROC (95% CI)	DOR (95% CI)	PLR (95% CI)	NLR (95% CI)
Study Design	cohort study	22	0.80[0.70,0.87]	0.80[0.72,0.85]	0.87[0.83–0.89]	16[9,29]	3.9[2.8,5.4]	0.25[0.16,0.38]
	case-control study	42	0.82[0.77,0.87]	0.83[0.78,0.87]	0.90[0.87–0.92]	23[13,40]	4.9[3.6,6.7]	0.21[0.16,0.29]
Determination method	ELISA	54	0.83[0.78,0.87]	0.83[0.78,0.86]	0.90[0.87–0.92]	23[15,36]	4.8[3.8,6.1]	0.21[0.16,0.27]
	IFA	1	0.81	0.74	NA	NA	NA	NA
	CMIA	2	0.75–0.89	0.73–0.95	NA	NA	NA	NA
	Immunoturbidimetry	7	0.82[0.73,0.88]	0.79[0.61,0.90]	0.86[0.82–0.88]	16[5,53]	3.8[1.8,7.9]	0.23[0.14,0.39]
Diagnostic criteria	pRIFLE	33	0.83[0.75,0.88]	0.85[0.79,0.89]	0.91[0.88–0.93]	27[14,52]	5.4[3.8,7.8]	0.20[0.14,0.30]
	AKIN	6	0.85[0.78,0.90]	0.88[0.82,0.92]	0.93[0.91–0.95]	43[22,85]	7.2[4.7,11.1]	0.17[0.11,0.25]
	KDIGO	25	0.79[0.72,0.84]	0.76[0.69,0.81]	0.84[0.81–0.87]	12[7,19]	3.3[2.5,4.2]	0.28[0.21,0.38]
Pathogenesis background	CPB	21	0.85[0.74,0.92]	0.88[0.83,0.92]	0.93[0.90–0.95]	41[17,97]	7.1[4.8,10.4]	0.17[0.10,0.30]
	Asphyxiated Neonates	15	0.83[0.77,0.88]	0.80[0.70,0.87]	0.88[0.85–0.91]	20[9,43]	4.1[2.6,6.6]	0.21[0.15,0.30]
	Critically ill children	17	0.72[0.63,0.80]	0.74[0.63,0.82]	0.79[0.75–0.82]	7[4,14]	2.7[1.9,4.1]	0.37[0.27,0.53]
	Critically-ill Neonates	11	0.83[0.74,0.90]	0.81[0.73,0.88]	0.89[0.86–0.92]	22[12,40]	4.5[3.1,6.5]	0.20[0.13,0.32]
Hospital departments	Admission	20	0.83[0.75,0.89]	0.84[0.77,0.90]	0.91[0.88–0.93]	27[11,62]	5.3[3.4,8.4]	0.20[0.13,0.32]
	NICU	27	0.83[0.79,0.87]	0.80[0.74,0.85]	0.89[0.86–0.91]	21[13,32]	4.3[3.2,5.7]	0.21[0.16,0.27]
	PICU	17	0.75[0.61,0.85]	0.82[0.71,0.89]	0.86[0.82–0.88]	13[6,31]	4.1[2.5,6.6]	0.31[0.19,0.50]
Age stage	Neonates	28	0.85[0.80,0.90]	0.82[0.76,0.87]	0.91[0.88–0.93]	27[15,49]	4.9[3.5,6.7]	0.18[0.13,0.25]
	children	36	0.78[0.71,0.84]	0.82[0.76,0.87]	0.87[0.84–0.90]	17[9,29]	4.4[3.1,6.1]	0.26[0.19,0.36]
Overall		64^a^	0.82[0.77,0.86]	0.82[0.78,0.86]	0.89[0.86–0.91]	21[14,31]	4.6[3.6,5.8]	0.22[0.17,0.29]

^a^ Since several included studies were multi-armed diagnostic tests, they also met our exclusion criteria. For each arm, it also met our inclusion criteria, so its overall number would outnumber the number of studies.

**Figure 3:**
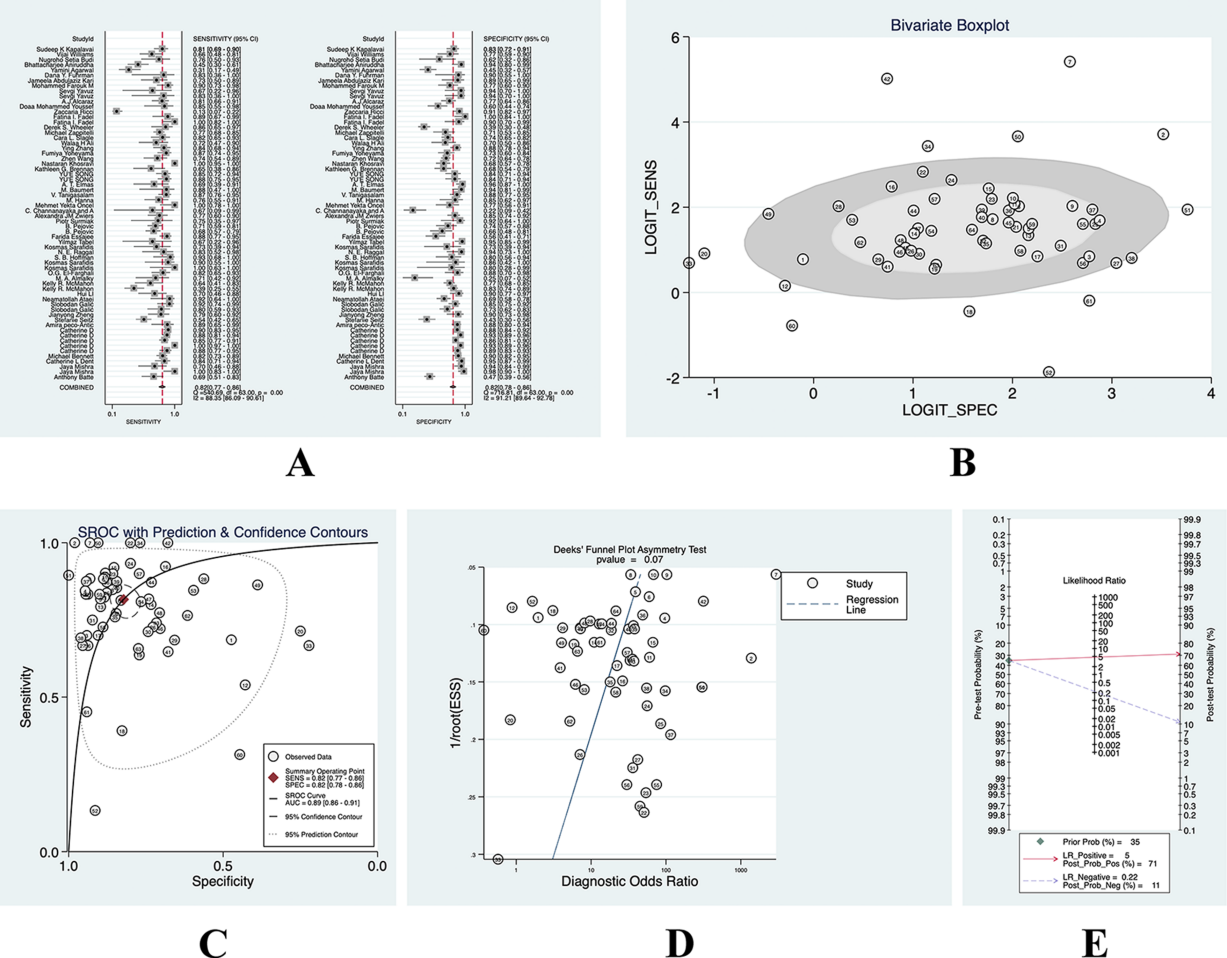
(**A**) forest plot of overall sensitivity and specificity; (**B**) boxplot of overall heterogeneity; (**C**) overall SROC curve; (**D**) overall deeks funnel chart; (**E**) nomogram of overall clinical utility.

We found that AKI occurred in 35% of these included patients, and we used its incidence as the prior probability. With a PLR of 5 (95% CI: 3.6–5.8), the probability of being diagnosed with AKI by a diagnostic test was 0.71 and the probability of being diagnosed with non-AKI was 0.11, and we found that there was no significant publication bias. (See Figure 3 for details).

#### 3.4.2. Prediction of AKI in children by NGAL in blood

After summing up all studies that measured blood NGAL, 22 studies on blood NGAL for predicting AKI were found. A bivariate effect model was used for the analysis, and the results are shown in Supplementary Table S2. The sensitivity was 0.79 (95% CI: 0.69–0.86), the specificity was 0.85 (95% CI: 0.75–0.91), and the SROC was 0.89 (95% CI: 0.89). 0.86–0.91), DOR 21 (95% CI: 9–48), PLR 5.1 (95% CI: 3.0–8.6), and NLR 0.25 (95% CI: 0.16–0.39).

**Figure 4:**
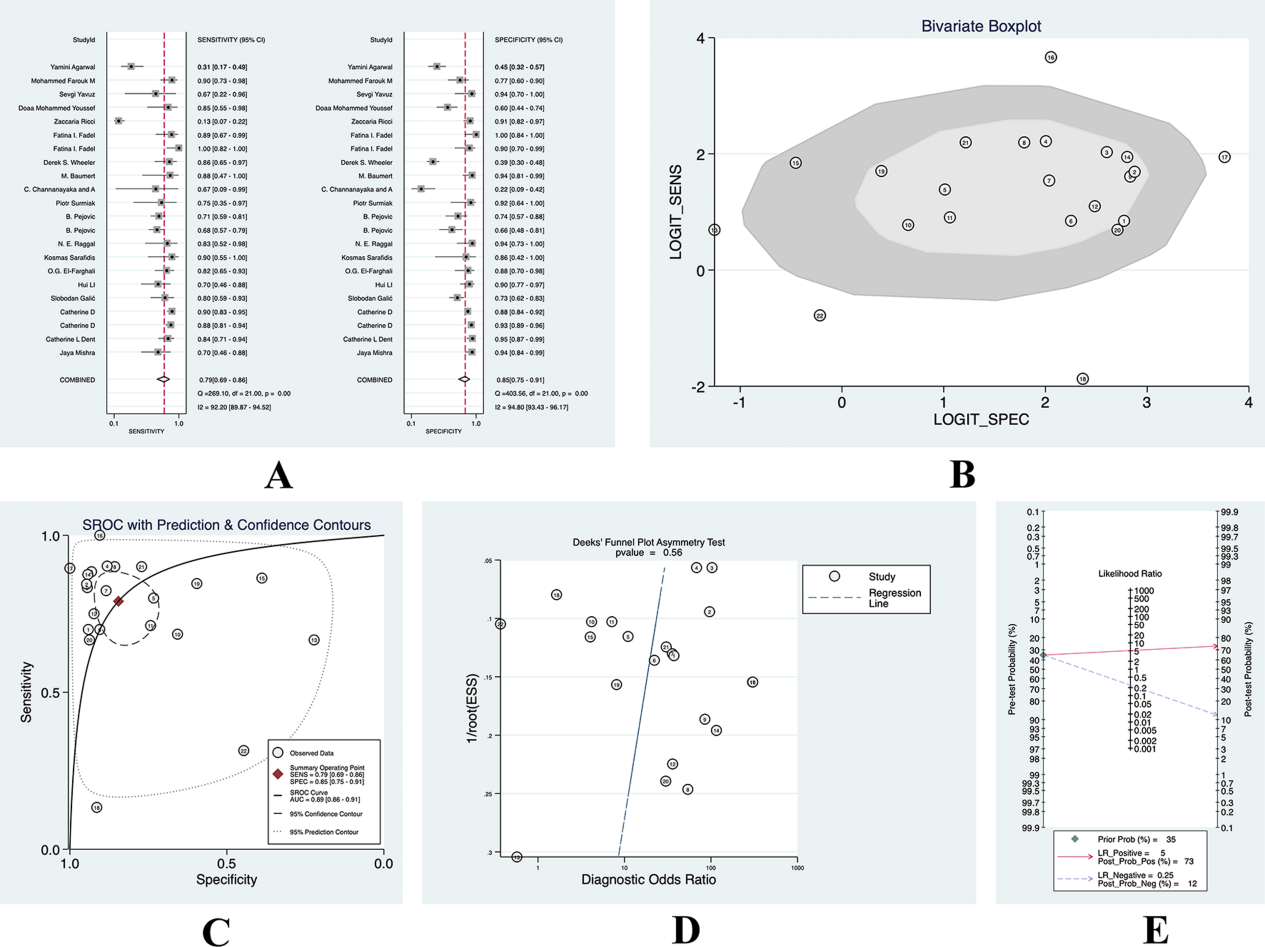
(**A**) sensitivity-specificity forest plot of blood NGAL; (**B**) heterogeneity—polygonal boxplot of blood NGAL; (**C**) SROC curve of blood NGAL; (**D**) deeks funnel diagram of blood NGAL; (**E**) clinical utility nomogram of blood NGAL.

Because the overall incidence of AKI of the patients we included was 35%, we still used the incidence of 35% as the prior probability. When the PLR was 5.1 (95% CI: 3.0–8.6), the probability of being diagnosed with AKI was 0.73 based on diagnostic tests and the probability of non-AKI was 0.12. See Figure 4 for details.

#### 3.4.3. Prediction of AKI in children by NGAL in urine

After summing all studies that measured urine NGAL, a total of 42 studies on urine NGAL for predicting AKI were found. The bivariate effect model was also used for analysis. The results are shown in Supplementary Table S3. The sensitivity was 0.83 (95% CI: 0.78–0.87), the specificity was 0.81 (95% CI: 0.77–0.85), and the SROC was 0.89 (95% CI: 0.86–0.91), DOR 21 (95% CI: 13–33), PLR 4.4 (95% CI: 3.5–5.6), and NLR 0.21 (95% CI: 0.16–0.28).

**Figure 5:**
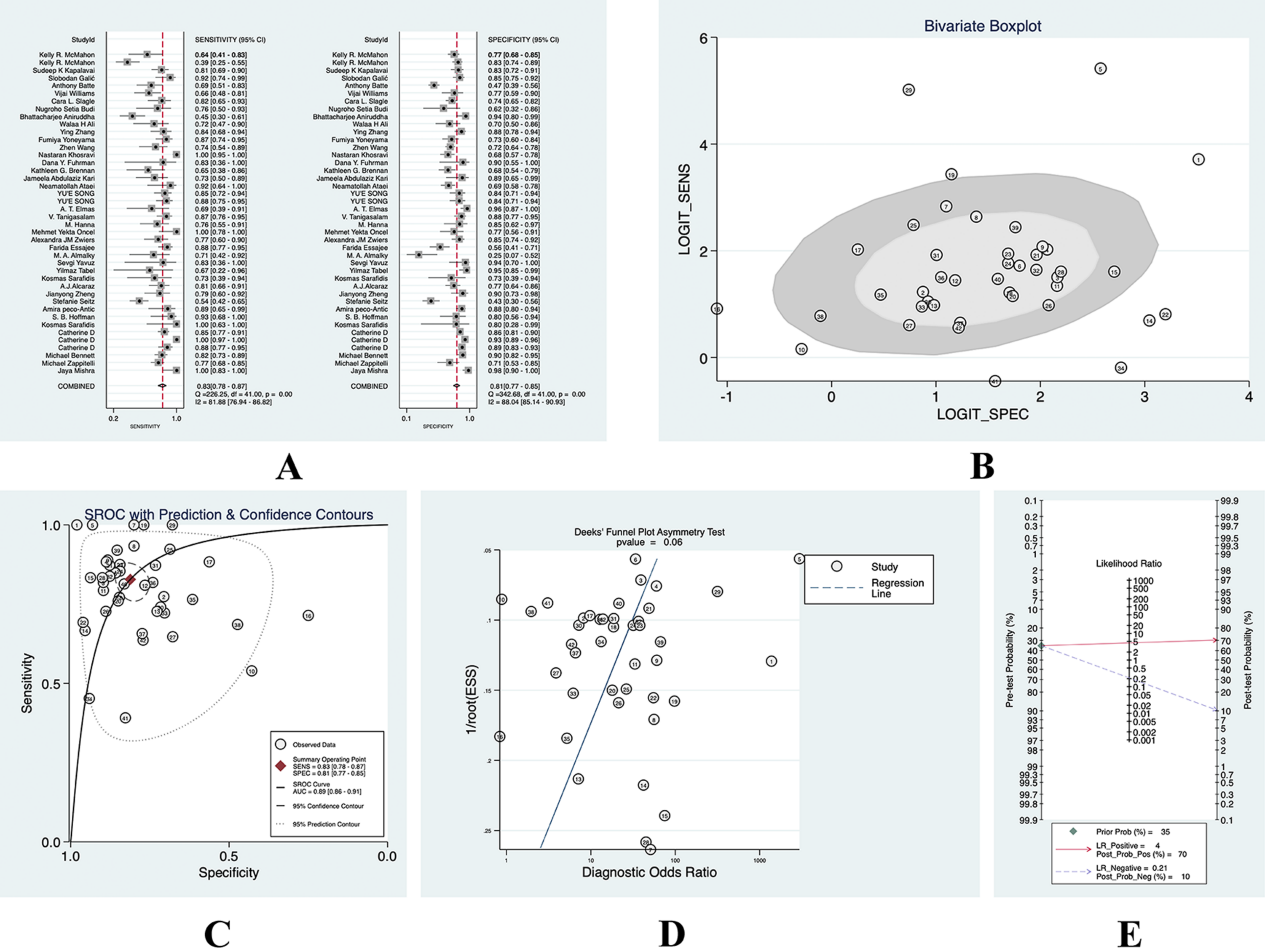
(**A**) sensitivity-specificity forest plot of urine NGAL; (**B**) heterogeneity—polygonal boxplot of urine NGAL; (**C**) SROC curve of urine NGAL; (**D**) deeks funnel diagram of urine NGAL; (**E**) clinical utility nomogram of urine NGAL.

We still adopted the 35% incidence of AKI as the prior probability. When the PLR was 4.4 (95% CI: 3.0–8.6), the probability of being diagnosed with AKI by diagnostic tests was 0.70, and the probability of being diagnosed as non-AKI was 0.10. See Figure 5.

#### 3.4.4. Measurement time

In the included studies, the urine or blood used to measure NGAL was collected at different times in different AKI backgrounds. For children after CPB surgery, the measurement time of NGAL was mainly in 2 h after surgery. The meta-analysis of the diagnostic accuracy reported a sensitivity of 0.90 (95% CI: 0.80–0.95), a specificity of 0.90 (95% CI: 0.83–0.94), SROC of 0.96 (95% CI: 0.93–0.97). For asphyxiated neonates, the measurement time of NGAL was mainly within 24 h after birth. The meta-analysis of the diagnostic tests reported a sensitivity of 0.88 (95% CI: 0.82–0.92), a specificity of 0.81 (95% CI: 0.71–0.90), SROC of 0.90 (95% CI: 0.87–0.92). After meta-analyzing all studies on the measurement time of NGAL after CPB and asphyxiated neonates, we found that the overall measurement accuracy was not as high as 2 h after CPB and 24 h after birth in asphyxiated neonates. The latter seemed to have a more ideal DOR, so we suggested that these time points were the reasonable sampling for AKI in both cases. See Table 3.

**Table 3:** Meta-analysis (sensitivity analysis) of NGAL in predicting AKI after CPB.

Subgroup	Number	Sensitivity (95% CI)	Specificity (95% CI)	SROC (95% CI)	DOR (95% CI)	PLR (95% CI)	NLR (95% CI)
After CPB (hours)	/	/	/	/	/	/	/
1	1	0.82	0.76	NA	NA	NA	NA
2	11	0.90[0.80,0.95]	0.90[0.83,0.94]	0.96[0.93–0.97]	80[24,269]	9.0[5.0,16.1]	0.11[0.05,0.23]
4	3	0.63–0.87	0.68–0.89	NA	NA	NA	NA
6	2	0.45–0.95	0.85–0.95	NA	NA	NA	NA
12	2	0.8–0.89	0.73–1	NA	NA	NA	NA
24	2	0.13–0.91	0.88–0.91	NA	NA	NA	NA
Overall	21	0.85[0.74,0.92]	0.88[0.83,0.92]	0.93[0.90–0.95]	41[17,97]	7.1[4.8,10.4]	0.17[0.10,0.30]
Asphyxiated Neonates (after birth, hours)	/	/	/	/	/	/	/
0	1	0.8	0.895	NA	NA	NA	NA
2	1	0.69	0.657	NA	NA	NA	NA
4	1	0.719	0.743	NA	NA	NA	NA
6	4	0.8	0.895	NA	NA	NA	NA
24	7	0.88[0.82,0.92]	0.82[0.71,0.90]	0.90[0.87–0.92]	34[16,72]	4.9[2.9,8.5]	0.15[0.10,0.22]
48	1	0.875	0.843	NA	NA	NA	NA
Overall	15	0.83[0.77,0.88]	0.80[0.70,0.87]	0.88[0.85–0.91]	20[9,43]	4.1[2.6,6.6]	0.21[0.15,0.30]

## 4. Discussion

We found NGAL had a high predictive value for AKI in children. There were no significant differences between blood and urine NGAL. At the same time, we also found that the measurement time of NGAL was mainly 2 h after CPB, and the main measurement time for asphyxiated neonates was 24 h after birth.

L. Taddeo et al. (70) conducted a systematic review in 2017 to evaluate the accuracy of NGAL which was taken as the biomarker of AKI in children. It analyzed a total of 13 studies including 1,629 children, and its combined sensitivity for urine NGAL was 0.76 (95% CI: 0.62–0.85), the combined specificity was 0.93 (95% CI: 0.88–0.96). When the number of included studies increased to 53 and the number of cases increased to 5,048 patients, the results of this study consist with those of previous studies.

We also found reports on other AKI predictors in recent years. H. M. Jia et al. (71) conducted a systematic review evaluating the effects of urinary tissue inhibitor of metalloproteinase-2 [TIMP-2] and insulin-like growth factor-binding protein 7 [IGFBP7] on the early diagnostic value of acute kidney injury, which included 9 eligible published studies with 1,886 cases. The combined sensitivity was 0.83 (95% CI: 0.79–0.87), and the specificity was 0.55 (95% CI: 0.52–0.57). We noticed that these two predictors had high predictive accuracy for the occurrence of AKI, however, its accuracy for the people without AKI was not enough. Compared with these two factors, NGAL had significantly improved sensitivity and specificity in predicting AKI. M. Fazel et al. (72) conducted a systematic review to assess the accuracy of Kidney Injury Molecule-1 (Kim-1) in predicting AKI in children within 12 h after admission, including 13 articles, which showed that the final AUC of urinary Kim-1 in predicting AKI was 0.69 (95% CI: 0.62–0.77), and its conclusion also suggested that Kim-1 seemed to have a moderate value in predicting AKI in children. P. Susantitaphong et al. (9) conducted a meta-analysis on 7 cohort studies. For urinary L-FABP, the sensitivity of for the diagnosis of AKI was 74.5% (95% CI: 60.4%–84.8%), and the specificity was 77.6% (95% CI: 61.5%–88.2%). In general, the current predictors of AKI in children include TIMP-2, IGFBP7, Kim-1, L-FABP, etc. Our study shows that NGAL seems to have a better predictive effect.

NGAL has different predictive accuracy in different AKI occurrence backgrounds. A. Haase-Fielitz et al. (73) conducted a systematic review that included 2,527 adults and 1,342 children who had undergone cardiac surgery. The results showed that urine NGAL could be used for the early prediction of AKI after cardiac surgery, but it did not carry out a meta-analysis, nor did it delve into the occurrence time of AKI and the measurement time of NGAL after cardiac surgery. F. F. Zhou et al. (74) researched on the diagnostic accuracy of NGAL within 12 h after cardiac surgery-associated acute kidney injury (CSA-AKI), including 24 studies (with 33 datasets of 4,066 patients). The overall sensitivity was 0.68 (95% CI: 0.65–0.70), and the specificity was 0.79 (95% CI: 0.77–0.80). After we expanded the number of included studies and the number of patients, we found that NGAL measurement performed 2 h after CPB had the best diagnostic performance for AKI.

Bellos et al. (75) conducted a systematic review of the accuracy of serum and urine NGAL in detecting AKI in neonates with perinatal asphyxia. A total of 11 studies were included, with a total of 652 cases. The sensitivity of serum NGAL in this study was 0.818 (95% CI: 0.668–0.909), and the specificity was 0.870 (95% CI: 0.754–0.936). The results of it are consistent with our results in the subgroup analysis of AKI associated with asphyxiated neonates, but this study did not conduct an analysis or draw conclusions on the best time for NGA1 measurement in asphyxiated neonates, so our study filled this gap.

This systematic review has the following advantages: (1) On the basis of previous studies, it comprehensively explored the predictive value of NGAL for children with AKI based on the results of many studies in recent years. (2) Among the newly discovered predictors of AKI in children in recent years, no matter it is from blood or urine, NGAL is a valuable predictor, which can provide a basis for the future clinical measurement of AKI, so that predictor samples can be collected through a non-invasively way and reduce patients' pain. (3) It is even found that there is no significant difference in various measurement methods, which can reduce the waste of medical resources caused by different measurement methods in clinical practice. (4) In addition, this study can also provide a reasonable choice of sampling or measurement time for predicting certain diseases leading to AKI. Effective predictors are essential for improving the predictive accuracy of risk models. Our research shows that NGAL has a good predictive value and can be included into the risk stratification models based on machine learning to improve their predictive accuracy.

However, we noticed some limitations. First, there was huge discrepancy between the number of studies which measure blood, urinary or both NGAL. Second, in the analyzed studies, there is various time gap between trial and gold standard analysis, and the AKI definition is also variable. Third, for the measurement time of some AKI backgrounds (such as sepsis, burns, solid tumors, use of nephrotoxic drugs, angiography-related, neonatal general surgery, etc.), due to the limited number of relevant reports, it may still be cautious to conclude in this regard.

## 5. Conclusion

NGAL is a valuable predictor for AKI in children with different backgrounds, and there is no significant difference in the predictive value of NGAL in urine and blood, which can provide a non-invasive choice for clinical practice. According to the current evidence, the accuracy of NGAL measurement is the best at 2 h after CPB and 24 h after birth in asphyxiated newborns. At the same time, we also noticed that there is no significant difference in different measurement methods of NGAL, which can provide guidance for the development and verification of assessment tools for the risk of AKI in children in the future. This variable can also be introduced into the machine learning method, and in the future, it is expected to develop a popular and clinically practical simple prediction system for the prediction of AKI in children based on the machine learning method.

## Data Availability

The original contributions presented in the study are included in the article/**Supplementary Material**, further inquiries can be directed to the corresponding author/s.
