# Supplementary material for: Predictive value of neutrophil gelatinase-associated lipocalin in children with acute kidney injury: A systematic review and meta-analysis
Source: Front Pediatr. 2023 Mar 27;11:1147033. doi: 10.3389/fped.2023.1147033 (PMC10083323; doi:10.3389/fped.2023.1147033)
Supplement: Supplementary file 1 [file Datasheet1.docx]

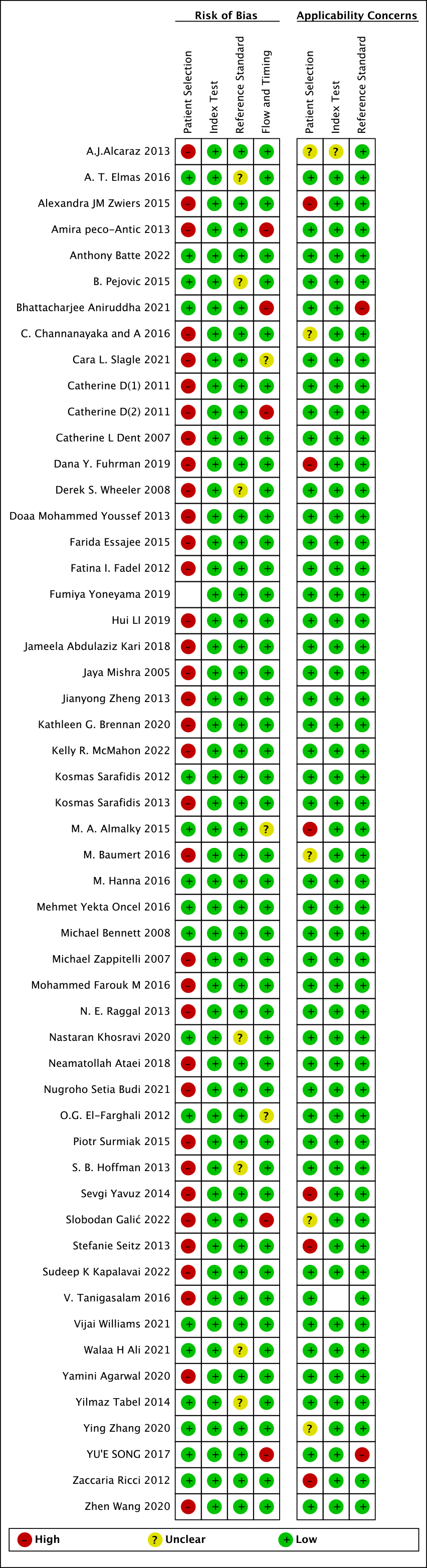


**Figure S1 |** Risk of bias summary

**TABLE S1 |** Literature search strategy

**1.Pubmed**

| Search number | Query | Results |
| --- | --- | --- |
| #1 | "Acute Kidney Injury"[Mesh] | 53,526 |
| #2 | ((((((((((((((((((((((((((Acute Kidney Injury[Title/Abstract]) OR (Acute Kidney Injuries[Title/Abstract])) OR (Kidney Injuries, Acute[Title/Abstract])) OR (Kidney Injury, Acute[Title/Abstract])) OR (Acute Renal Injury[Title/Abstract])) OR (Acute Renal Injuries[Title/Abstract])) OR (Renal Injuries, Acute[Title/Abstract])) OR (Renal Injury, Acute[Title/Abstract])) OR (Renal Insufficiency, Acute[Title/Abstract])) OR (Acute Renal Insufficiencies[Title/Abstract])) OR (Renal Insufficiencies, Acute[Title/Abstract])) OR (Acute Renal Insufficiency[Title/Abstract])) OR (Kidney Insufficiency, Acute[Title/Abstract])) OR (Acute Kidney Insufficiencies[Title/Abstract])) OR (Kidney Insufficiencies, Acute[Title/Abstract])) OR (Acute Kidney Insufficiency[Title/Abstract])) OR (Kidney Failure, Acute[Title/Abstract])) OR (Acute Kidney Failures[Title/Abstract])) OR (Kidney Failures, Acute[Title/Abstract])) OR (Acute Renal Failure[Title/Abstract])) OR (Acute Renal Failures[Title/Abstract])) OR (Renal Failures, Acute[Title/Abstract])) OR (Renal Failure, Acute[Title/Abstract])) OR (Acute Kidney Failure[Title/Abstract])) OR (acute kidney insufficiency[Title/Abstract])) OR (kidney acute failure[Title/Abstract]) | 73,996 |
| #3 | ("Acute Kidney Injury"[Mesh]) OR (((((((((((((((((((((((((((Acute Kidney Injury[Title/Abstract]) OR (Acute Kidney Injuries[Title/Abstract])) OR (Kidney Injuries, Acute[Title/Abstract])) OR (Kidney Injury, Acute[Title/Abstract])) OR (Acute Renal Injury[Title/Abstract])) OR (Acute Renal Injuries[Title/Abstract])) OR (Renal Injuries, Acute[Title/Abstract])) OR (Renal Injury, Acute[Title/Abstract])) OR (Renal Insufficiency, Acute[Title/Abstract])) OR (Acute Renal Insufficiencies[Title/Abstract])) OR (Renal Insufficiencies, Acute[Title/Abstract])) OR (Acute Renal Insufficiency[Title/Abstract])) OR (Kidney Insufficiency, Acute[Title/Abstract])) OR (Acute Kidney Insufficiencies[Title/Abstract])) OR (Kidney Insufficiencies, Acute[Title/Abstract])) OR (Acute Kidney Insufficiency[Title/Abstract])) OR (Kidney Failure, Acute[Title/Abstract])) OR (Acute Kidney Failures[Title/Abstract])) OR (Kidney Failures, Acute[Title/Abstract])) OR (Acute Renal Failure[Title/Abstract])) OR (Acute Renal Failures[Title/Abstract])) OR (Renal Failures, Acute[Title/Abstract])) OR (Renal Failure, Acute[Title/Abstract])) OR (Acute Kidney Failure[Title/Abstract])) OR (acute kidney insufficiency[Title/Abstract])) OR (kidney acute failure[Title/Abstract])) | 88,347 |
| #4 | "Infant, Newborn"[Mesh] | 656,979 |
| #5 | ((((((((Infant, Newborn[Title/Abstract]) OR (Newborn Infant[Title/Abstract])) OR (Newborn Infants[Title/Abstract])) OR (Newborns[Title/Abstract])) OR (Neonate[Title/Abstract])) OR (Neonates[Title/Abstract])) OR (newborn[Title/Abstract])) OR (newly born baby[Title/Abstract])) OR (newly born child[Title/Abstract]) | 275,446 |
| #6 | ("Infant, Newborn"[Mesh]) OR (((((((((Infant, Newborn[Title/Abstract]) OR (Newborn Infant[Title/Abstract])) OR (Newborn Infants[Title/Abstract])) OR (Newborns[Title/Abstract])) OR (Neonate[Title/Abstract])) OR (Neonates[Title/Abstract])) OR (newborn[Title/Abstract])) OR (newly born baby[Title/Abstract])) OR (newly born child[Title/Abstract])) | 757,674 |
| #7 | "Infant"[Mesh] | 1,224,631 |
| #8 | (Infant[Title/Abstract]) OR (Infants[Title/Abstract]) | 449,572 |
| #9 | ("Infant"[Mesh]) OR ((Infant[Title/Abstract]) OR (Infants[Title/Abstract])) | 1,329,125 |
| #10 | "Child"[Mesh] | 2,089,754 |
| #11 | (Child[Title/Abstract]) OR (Children[Title/Abstract]) | 1,433,662 |
| #12 | ("Child"[Mesh]) OR ((Child[Title/Abstract]) OR (Children[Title/Abstract])) | 2,527,957 |
| #13 | ((("Infant, Newborn"[Mesh]) OR (((((((((Infant, Newborn[Title/Abstract]) OR (Newborn Infant[Title/Abstract])) OR (Newborn Infants[Title/Abstract])) OR (Newborns[Title/Abstract])) OR (Neonate[Title/Abstract])) OR (Neonates[Title/Abstract])) OR (newborn[Title/Abstract])) OR (newly born baby[Title/Abstract])) OR (newly born child[Title/Abstract]))) OR (("Infant"[Mesh]) OR ((Infant[Title/Abstract]) OR (Infants[Title/Abstract])))) OR (("Child"[Mesh]) OR ((Child[Title/Abstract]) OR (Children[Title/Abstract]))) | 3,186,977 |
| #14 | (((neutrophil gelatinase-associated lipocalin[Title/Abstract]) OR (NGAL[Title/Abstract])) OR (uNGAL[Title/Abstract])) OR (neutrophil gelatinase associated lipocalin[Title/Abstract]) | 4,625 |
| #15 | ((("Acute Kidney Injury"[Mesh]) OR (((((((((((((((((((((((((((Acute Kidney Injury[Title/Abstract]) OR (Acute Kidney Injuries[Title/Abstract])) OR (Kidney Injuries, Acute[Title/Abstract])) OR (Kidney Injury, Acute[Title/Abstract])) OR (Acute Renal Injury[Title/Abstract])) OR (Acute Renal Injuries[Title/Abstract])) OR (Renal Injuries, Acute[Title/Abstract])) OR (Renal Injury, Acute[Title/Abstract])) OR (Renal Insufficiency, Acute[Title/Abstract])) OR (Acute Renal Insufficiencies[Title/Abstract])) OR (Renal Insufficiencies, Acute[Title/Abstract])) OR (Acute Renal Insufficiency[Title/Abstract])) OR (Kidney Insufficiency, Acute[Title/Abstract])) OR (Acute Kidney Insufficiencies[Title/Abstract])) OR (Kidney Insufficiencies, Acute[Title/Abstract])) OR (Acute Kidney Insufficiency[Title/Abstract])) OR (Kidney Failure, Acute[Title/Abstract])) OR (Acute Kidney Failures[Title/Abstract])) OR (Kidney Failures, Acute[Title/Abstract])) OR (Acute Renal Failure[Title/Abstract])) OR (Acute Renal Failures[Title/Abstract])) OR (Renal Failures, Acute[Title/Abstract])) OR (Renal Failure, Acute[Title/Abstract])) OR (Acute Kidney Failure[Title/Abstract])) OR (acute kidney insufficiency[Title/Abstract])) OR (kidney acute failure[Title/Abstract]))) AND (((("Infant, Newborn"[Mesh]) OR (((((((((Infant, Newborn[Title/Abstract]) OR (Newborn Infant[Title/Abstract])) OR (Newborn Infants[Title/Abstract])) OR (Newborns[Title/Abstract])) OR (Neonate[Title/Abstract])) OR (Neonates[Title/Abstract])) OR (newborn[Title/Abstract])) OR (newly born baby[Title/Abstract])) OR (newly born child[Title/Abstract]))) OR (("Infant"[Mesh]) OR ((Infant[Title/Abstract]) OR (Infants[Title/Abstract])))) OR (("Child"[Mesh]) OR ((Child[Title/Abstract]) OR (Children[Title/Abstract]))))) AND ((((neutrophil gelatinase-associated lipocalin[Title/Abstract]) OR (NGAL[Title/Abstract])) OR (uNGAL[Title/Abstract])) OR (neutrophil gelatinase associated lipocalin[Title/Abstract])) | 306 |

**2.Cochrane**

| Search number | Query | Results |
| --- | --- | --- |
| #1 | MeSH descriptor: [Acute Kidney Injury] explode all trees | 1688 |
| #2 | (Acute Kidney Injury):ti,ab,kw OR (Acute Kidney Injuries):ti,ab,kw OR (Kidney Injuries, Acute):ti,ab,kw OR (Kidney Injury, Acute):ti,ab,kw OR (Acute Renal Injury):ti,ab,kw | 5092 |
| #3 | (Acute Renal Injuries):ti,ab,kw OR (Renal Injuries, Acute):ti,ab,kw OR (Renal Injury, Acute):ti,ab,kw OR (Renal Insufficiency, Acute):ti,ab,kw OR (Acute Renal Insufficiencies):ti,ab,kw | 4013 |
| #4 | (Renal Insufficiencies, Acute):ti,ab,kw OR (Acute Renal Insufficiency):ti,ab,kw OR (Kidney Insufficiency, Acute):ti,ab,kw OR (Acute Kidney Insufficiencies):ti,ab,kw OR (Kidney Insufficiencies, Acute):ti,ab,kw | 1191 |
| #5 | (Acute Kidney Insufficiency):ti,ab,kw OR (Kidney Failure, Acute):ti,ab,kw OR (Acute Kidney Failures):ti,ab,kw OR (Kidney Failures, Acute):ti,ab,kw OR (Acute Renal Failure):ti,ab,kw | 8579 |
| #6 | (Acute Renal Failures):ti,ab,kw OR (Renal Failures, Acute):ti,ab,kw OR (Renal Failure, Acute):ti,ab,kw OR (acute kidney failure):ti,ab,kw OR (kidney acute failure):ti,ab,kw | 8308 |
| #7 | #1 or #2 or #3 or #4 or #5 or #6 | 10674 |
| #8 | MeSH descriptor: [Infant, Newborn] explode all trees | 17700 |
| #9 | (Infant, Newborn):ti,ab,kw OR (Infants, Newborn):ti,ab,kw OR (Newborn Infant):ti,ab,kw OR (Newborn Infants):ti,ab,kw OR (Newborns):ti,ab,kw | 26243 |
| #10 | (Neonate):ti,ab,kw OR (Neonates):ti,ab,kw OR (newborn):ti,ab,kw OR (newly born baby):ti,ab,kw OR (newly born child):ti,ab,kw | 33264 |
| #11 | #8 or #9 or #10 | 34702 |
| #12 | MeSH descriptor: [Infant] explode all trees | 35185 |
| #13 | (Infant):ti,ab,kw OR (Infants):ti,ab,kw | 66769 |
| #14 | #12 or #13 | 66769 |
| #15 | MeSH descriptor: [Child] explode all trees | 61999 |
| #16 | (Child):ti,ab,kw OR (Children):ti,ab,kw | 166709 |
| #17 | #15 or #16 | 166709 |
| #18 | #11 or #14 or #17 | 207573 |
| #19 | (neutrophil gelatinase associated lipocalin):ti,ab,kw OR (NGAL):ti,ab,kw OR (uNGAL):ti,ab,kw OR (neutrophil gelatinase-associated lipocalin):ti,ab,kw | 926 |
| #20 | #7 and #18 and #19 | 39 |

**3.Embase**

| Search number | Query | Results |
| --- | --- | --- |
| #1 | acute kidney failure'/exp | 113901 |
| #2 | acute kidney failure':ab,ti OR 'acute kidney injury':ab,ti OR 'acute kidney injuries':ab,ti OR 'kidney injuries, acute':ab,ti OR 'kidney injury, acute':ab,ti OR 'acute renal injury':ab,ti OR 'acute renal injuries':ab,ti OR 'renal injuries, acute':ab,ti OR 'renal injury, acute':ab,ti OR 'renal insufficiency, acute':ab,ti OR 'acute renal insufficiencies':ab,ti OR 'renal insufficiencies, acute':ab,ti OR 'acute renal insufficiency':ab,ti OR 'kidney insufficiency, acute':ab,ti OR 'acute kidney insufficiencies':ab,ti OR 'kidney insufficiencies, acute':ab,ti OR 'acute kidney insufficiency':ab,ti OR 'kidney failure, acute':ab,ti OR 'acute kidney failures':ab,ti OR 'kidney failures, acute':ab,ti OR 'acute renal failure':ab,ti OR 'acute renal failures':ab,ti OR 'renal failures, acute':ab,ti OR 'renal failure, acute':ab,ti OR 'kidney acute failure':ab,ti | 90156 |
| #3 | #1 OR #2 | 130838 |
| #4 | newborn'/exp | 650099 |
| #5 | newborn:ab,ti OR 'infant, newborn':ab,ti OR 'infants, newborn':ab,ti OR 'newborn infant':ab,ti OR 'newborn infants':ab,ti OR newborns:ab,ti OR neonate:ab,ti OR neonates:ab,ti OR 'newly born baby':ab,ti OR 'newly born child':ab,ti | 337686 |
| #6 | #4 OR #5 | 762381 |
| #7 | infant'/exp | 1239262 |
| #8 | infant:ab,ti OR infants:ab,ti | 499451 |
| #9 | #7 OR #8 | 1389952 |
| #10 | child'/exp | 3209218 |
| #11 | child:ab,ti OR children:ab,ti | 1843092 |
| #12 | #10 OR #11 | 3646191 |
| #13 | #6 OR #9 OR #12 | 3795255 |
| #14 | neutrophil gelatinase associated lipocalin'/exp | 11457 |
| #15 | neutrophil gelatinase associated lipocalin':ab,ti OR ungal:ab,ti OR ngal:ab,ti OR 'neutrophil gelatinase-associated lipocalin':ab,ti | 7860 |
| #16 | #14 OR #15 | 12928 |
| #17 | #3 AND #13 AND #16 | 529 |

**4.Web of science**

| Search number | Query | Results |
| --- | --- | --- |
| #1 | Acute Kidney Injury (Topic) OR Acute Kidney Injuries (Topic) OR Kidney Injuries, Acute (Topic) OR Kidney Injury, Acute (Topic) OR Acute Renal Injury (Topic) OR Acute Renal Injuries (Topic) OR Renal Injuries, Acute (Topic) OR Renal Injury, Acute (Topic) OR Renal Insufficiency, Acute (Topic) OR Acute Renal Insufficiencies (Topic) OR Renal Insufficiencies, Acute (Topic) OR Acute Renal Insufficiency (Topic) OR Kidney Insufficiency, Acute (Topic) OR Acute Kidney Insufficiencies (Topic) OR Kidney Insufficiencies, Acute (Topic) OR Acute Kidney Insufficiency (Topic) OR Kidney Failure, Acute (Topic) OR Acute Kidney Failures (Topic) OR Kidney Failures, Acute (Topic) OR Acute Renal Failure (Topic) OR Acute Renal Failures (Topic) OR Renal Failures, Acute (Topic) OR Renal Failure, Acute (Topic) OR acute kidney failure (Topic) OR kidney acute failure (Topic) | 95119 |
| #2 | Infant, Newborn (Topic) OR Infants, Newborn (Topic) OR Newborn Infant (Topic) OR Newborn Infants (Topic) OR Newborns (Topic) OR Neonate (Topic) OR Neonates (Topic) OR newborn (Topic) OR newly born baby (Topic) OR newly born child (Topic) OR Infant (Topic) OR Infants (Topic) OR Child (Topic) OR Children (Topic) | 2269793 |
| #3 | neutrophil gelatinase-associated lipocalin (Topic) OR NGAL (Topic) OR uNGAL (Topic) OR neutrophil gelatinase associated lipocalin (Topic) | 5781 |
| #4 | #3 AND #2 AND #1 | 424 |

**TABLE S2 |** Meta-analysis (sensitivity analysis) of predicting AKI by blood NGAL

| Subgroup | Levels | Number | Sensitivity(95% CI) | Specificity(95% CI) | SROC(95% CI) | DOR(95% CI) | PLR(95% CI) | NLR(95% CI) |
| --- | --- | --- | --- | --- | --- | --- | --- | --- |
| Study Design | cohort study | 5 | 0.64[0.35,0.86] | 0.81[0.68,0.89] | 0.82[0.78,0.85] | 7[3,21] | 3.3[2.1,5.3] | 0.44[0.21,0.91] |
|  | case-control study | 17 | 0.82[0.74,0.88] | 0.85[0.74,0.92] | 0.90[0.87,0.92] | 26[10,72] | 5.5[2.9,10.4] | 0.21[0.13,0.33] |
| Determination method | ELISA | 18 | 0.82[0.74,0.87] | 0.85[0.77,0.91] | 0.90[0.87-0.92] | 26[11,61] | 5.6[3.3,9.6] | 0.22[0.15,0.32] |
|  | IFA | 1 | 1 | 0.81 | 0.74 | NA | NA | NA |
|  | Immunoturbidimetry | 3 | 3 | 0.74-0.85 | 0.76-0.93 | NA | NA | NA |
| Diagnostic criteria | pRIFLE | 12 | 0.80[0.62,0.91] | 0.86[0.74,0.93] | 0.90[0.87-0.93] | 25[7,88] | 5.8[2.8,11.9] | 0.23[0.11,0.48] |
|  | AKIN | 3 |  |  |  |  |  |  |
|  | KDIGO | 7 | 0.73[0.66,0.79] | 0.76[0.55,0.89] | 0.76[0.73-0.80] | 9[3,27] | 3.0[1.4,6.6] | 0.36[0.24,0.54] |
| Pathogenesis background | CPB | 8 | 0.82[0.60,0.93] | 0.91[0.86,0.95] | 0.94[0.91-0.96] | 48[14,168] | 9.3[5.5,15.8] | 0.19[0.08,0.50] |
|  | Asphyxiated Neonates | 7 | 0.75[0.64,0.83] | 0.82[0.58,0.93] | 0.81[0.77-0.84] | 13[3,61] | 4.1[1.4,11.4] | 0.31[0.18,0.53] |
|  | Critically-ill children | 5 | 0.73[0.48,0.88] | 0.73[0.47,0.89] | 0.79[0.75-0.82] | 7[1,37] | 2.7[1.1,6.7] | 0.38[0.16,0.89] |
|  | Critically-ill  Neonates | 2 | 0.63-0.81 | 0.68-0.90 | NA | NA | NA | NA |
| Hospital departments | Admission | 5 | 0.82[0.73,0.89] | 0.89 [0.81,0.94] | 0.92[0.89-0.94] | 39[18,87] | 7.8 [4.3,13.9] | 0.20[0.13,0.31] |
|  | NICU | 9 | 0.79[0.70,0.86] | 0.84 [0.68,0.93] | 0.87[0.84-0.90] | 20 [5,76] | 4.9 [2.1,11.3] | 0.25 0.15,0.42] |
|  | PICU | 8 | 0.78[0.48,0.93] | 0.81[0.59,0.93] | 0.86[0.83-0.89] | 15[2,91] | 4.1[1.6,10.4] | 0.27[0.09,0.82] |
| Age stage | Neonates | 9 | 0.79[0.70,0.86] | 0.84[0.68,0.93] | 0.87[0.84-0.90] | 20[5,76] | 4.9[2.1,11.3] | 0.25[0.15,0.42] |
|  | children | 13 | 0.78[0.62,0.89] | 0.85[0.73,0.92] | 0.89[0.86-0.91] | 20[6,62] | 5.1[2.7,9.8] | 0.26[0.13,0.49] |
| Overall |  | 22 | 0.79[0.69,0.86] | 0.85[0.75,0.91] | 0.89[0.86-0.91] | 21[9,48] | 5.1[3.0,8.6] | 0.25[0.16,0.39] |

**TABLE S3 |** Meta-analysis (sensitivity analysis) of predicting AKI by urine NGAL

| Subgroup | Number | sensitivity(95% CI) | Specificity(95% CI) | SROC(95% CI) | DOR(95% CI) | PLR(95% CI) | NLR(95% CI) |
| --- | --- | --- | --- | --- | --- | --- | --- |
| Study Design | 25 | 0.83[0.75,0.88] | 0.82[0.77,0.86] | 0.89[0.86-0.92] | 22[12,43] | 4.7[3.5,6.3] | 0.21[0.14,0.31] |
|  | 17 | 0.83[0.76,0.88] | 0.79[0.70,0.86] | 0.88[0.85-0.91] | 18[10,35] | 3.9[2.7,5.8] | 0.21[0.15,0.31] |
| Determination method | 36 | 0.84[0.78,0.88] | 0.82[0.77,0.86] | 0.89[0.86-0.92] | 23[13,38] | 4.5[3.5,5.9] | 0.20[0.14,0.28] |
|  | 2 | 0.75-0.89 | 0.73-0.95 | NA | NA | NA | NA |
|  | 4 | 0.83[0.75,0.89] | 0.84[0.78,0.88] | 0.90[0.87-0.92] | 25[14,45] | 5.1[3.7,7.0] | 0.20[0.14,0.30] |
| Diagnostic criteria | 21 | 0.84[0.76,0.89] | 0.84[0.77,0.89] | 0.91[0.88-0.93] | 27[13,57] | 5.2[3.5,7.7] | 0.19[0.13,0.29] |
|  | 3 | 0.80-0.89 | 0.88-0.95 | NA | NA | NA | NA |
|  | 18 | 0.80[0.71,0.87] | 0.76[0.69,0.81] | 0.84[0.81-0.87] | 13[7,22] | 3.3[2.6,4.3] | 0.26[0.18,0.39] |
| Pathogenesis background | 13 | 0.86[0.74,0.93] | 0.85[0.78,0.90] | 0.92[0.89-0.94] | 35[11,109] | 5.8[3.5,9.4] | 0.16[0.08,0.33] |
|  | 8 | 0.87[0.82,0.90] | 0.80[0.72,0.87] | 0.88[0.85-0.90] | 27[15,47] | 4.4[3.0,6.3] | 0.16[0.12,0.22] |
|  | 12 | 0.72[0.63,0.79] | 0.74[0.63,0.82] | 0.79[0.75-0.82] | 7[4,14] | 2.8[1.9,4.0] | 0.38[0.28,0.52] |
|  | 9 | 0.83[0.70,0.91] | 0.83[0.74,0.89] | 0.89[0.86-0.92] | 23[12,46] | 4.8[3.2,7.0] | 0.21[0.12,0.37] |
| Hospital departments | 14 | 0.84[0.72,0.92] | 0.81[0.70,0.89] | 0.90[0.87-0.92] | 23[7,81] | 4.5[2.5,8.0] | 0.19[0.09,0.40] |
|  | 19 | 0.85[0.85,0.85] | 0.80[0.80,0.80] | 0.89[0.86-0.92] | 23[23,23] | 4.3[4.3,4.3] | 0.19[0.19,0.19] |
|  | 9 | 0.73[0.64,0.80] | 0.82[0.75,0.88] | 0.85[0.82-0.88] | 12[8,19] | 4.1[3.0,5.7] | 0.33[0.25,0.43] |
| Age stage | 19 | 0.88[0.80,0.93] | 0.82[0.76,0.87] | 0.91[0.88-0.93] | 32[16,64] | 4.9[3.6,6.5] | 0.15[0.09,0.25] |
|  | 23 | 0.78[0.71,0.84] | 0.81[0.73,0.86] | 0.86[0.83-0.89] | 15[8,28] | 4.0[2.8,5.8] | 0.27[0.20,0.38] |
| Overall | 42 | 0.83[0.78,0.87] | 0.81[0.77,0.85] | 0.89[0.86-0.91] | 21[13,33] | 4.4[3.5,5.6] | 0.21[0.16,0.28] |
